# Estimation of savings of life-years and cost from early detection of cervical cancer: a follow-up study using nationwide databases for the period 2002–2009

**DOI:** 10.1186/1471-2407-14-505

**Published:** 2014-07-10

**Authors:** Mei-Chuan Hung, Meng-Ting Liu, Ya-Min Cheng, Jung-Der Wang

**Affiliations:** 1 Department of Public Health, National Cheng Kung University College of Medicine, No.1, University Road, Tainan, Taiwan; 2 Department of Obstetrics and Gynecology, National Cheng Kung University Hospital, College of Medicine, National Cheng Kung University, 138 Sheng-Li Road, Tainan, Taiwan; 3 Departments of Internal Medicine and Occupational and Environmental Medicine, National Cheng Kung University Hospital, College of Medicine, National Cheng Kung University, Tainan, Taiwan

**Keywords:** Life-years, Cost, Early detection, Cervical cancer, Semi-parametric extrapolation

## Abstract

**Background:**

Few studies consider both the survival and financial benefits of detection of invasive cervical cancer (ICC) at earlier stages. This study estimated the savings in life-years and costs from early diagnosis of cervical cancer using an *ex post* approach.

**Methods:**

A total of 28,797 patients diagnosed with cervical cancer in the period 2002–2009 were identified from the National Cancer Registry of Taiwan, and linked to the National Mortality Registry until the end of 2011. Life expectancies (LE) for cancer at different stages were estimated using a semi-parametric extrapolation method. The expected years of life lost (EYLL) for cancer were calculated by subtracting the LE of the cancer cohort from that of the age-and sex-matched general population. The mean lifetime costs after diagnosis paid by the single-payer National Health Insurance during (NHI) 2002–2010 were estimated by multiplying average monthly expenditures by the survival probabilities and summing up over lifetime.

**Results:**

ICC at stages 1 to 4 had an average EYLL of 6.33 years, 11.64 years, 12.65 years, and 18.61 years, respectively, while the related lifetime costs paid by the NHI were $7,020, $10,133, $11,120, and $10,015 US dollars, respectively; the younger the diagnosis age, the higher the savings with regard to EYLL. The mean lifetime costs of managing cervical cancer were generally lower for the earlier stages compared with stages 3 and 4.

**Conclusions:**

Early detection of ICC saves lives and reduces healthcare costs. These health benefits and monetary savings can be used for cost-effectiveness assessments and the promotion of regular proactive screening, especially among older women.

## Background

Cervical cancer is one of the most prevalent types of cancer in women, with 530,232 annual cases and 275,008 deaths worldwide in 2008 [1]. Because of widespread screening programs coupled with advanced medical treatment technologies, women with cervical cancer now have relatively high five-year survival rates [2-5], and there is a consensus that early detection of cervical cancer can avoid premature mortality [3,4]. The Taiwanese government launched a nationwide cervical screening program in July 1995, in which annual pap smear screenings are offered to women aged over 30. Recent records from 2009 indicate that the compliance rate for pap testing in Taiwan is approximately 50% by age 65, which drops to 30.5% at age 70 or older [6]. If patients with invasive cancer could be detected at an earlier stage, the potential benefits with regard to the expected years of life lost (EYLL) [7] and healthcare expenditure would create additional incentives for cancer screening and treatment. Although there are quite a few studies which explore these issues [7-12], the question of how the various stages of cervical cancer at detection in different age groups influence outcomes in patient management remains less clear. In this study, the authors thus used an *ex post* approach based on national databases in Taiwan to estimate the life years and healthcare expenditures saved from early detection of cervical cancer, stratified by both stages and age.

## Methods

### Study population and datasets

The study commenced after the approval of the Institutional Review Board of National Cheng Kung University Hospital (NCKUH, IRB number: ER-102-034). A total of 28,797 patients diagnosed with cervical cancer in 2002–2009 were identified from the National Cancer Registry of Taiwan [13], which contains data on cancer staging, diagnosis date and age. The cancer site of interest is the cervix (ICD-9-CM code: 180). Gynecologists in Taiwan generally follow the clinical staging of FIGO (International Federation of Gynecology and Obstetrics) [14], and adopt the treatment guidelines of NCCN (National Comprehensive Cancer Network) for invasive cervical cancer [15]. The authors classified cervical cancer into stage 0 and invasive cancer (stages 1–4).

### Survival analysis and extrapolation to estimate life expectancy and EYLL stratified by stages in different age groups

**Figure 1:**
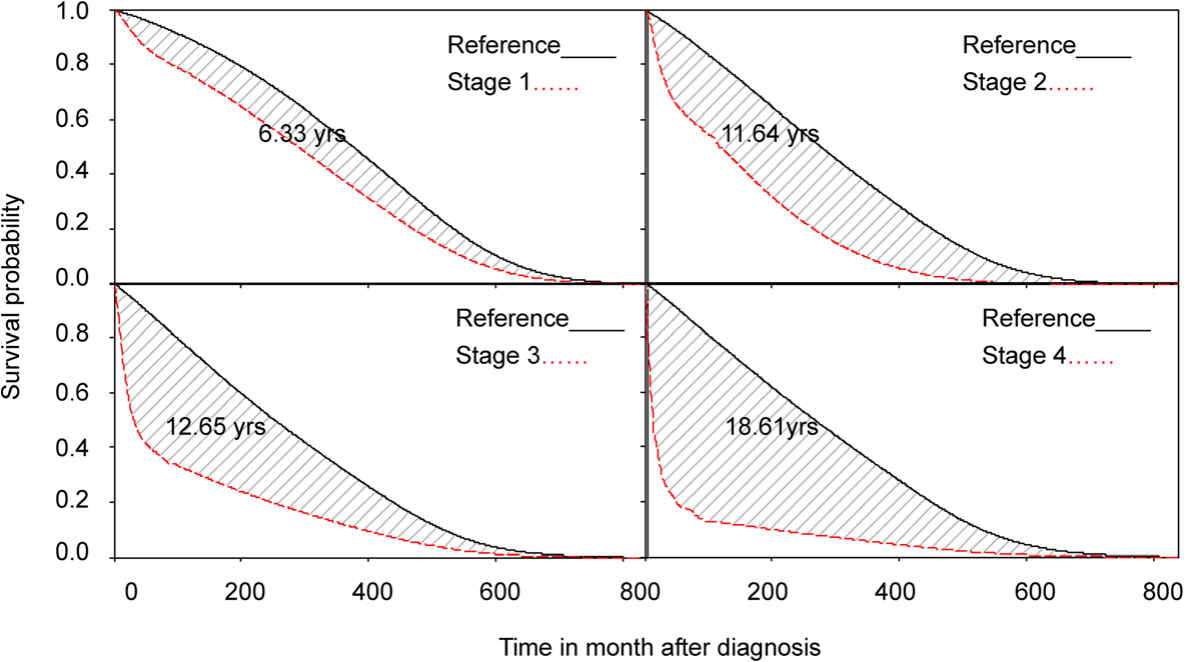
**Average expected years of life lost (EYLL) due to cervical cancer stratified by stages.** The difference (shadowed area) of LE between the cohort of cervical cancer and age-, gender-matched reference population, which represents the average EYLL of developing a case of cervical cancer.

All of the above patients were followed until the end of 2011 and linked with the National Mortality Registry to obtain the survival function via the Kaplan-Meier estimation method. They were further extrapolated to lifetime based on a semi-parametric method using the age- and sex-matched referents simulated from the life tables of the National Vital Statistics of Taiwan, which only requires an assumption of constant excess hazards [16,17]. The estimates were obtained using iSQoL software [18]. Detailed methods and mathematical proofs are described in our previous studies [7-9,16,17]. The average EYLL [7,9] for patients was calculated by the age- and gender-matched reference subjects simulated from the hazard functions of the vital statistics and subtracting the life expectancy of cancer patients, as shown in Figure 1. Z-tests were also performed for each group, with a p-value < .05 considered statistically significant.

### Lifetime cost paid by the National Health Insurance (NHI) stratified by stages in different age groups

This study estimated the lifetime cost by counting the monthly average dollars reimbursed by the NHI during 2002–2010 for these patients, from the day of validated diagnosis to the end of life or censored [19,20]. In general, the NHI comprehensively reimburses all medical services for each cancer patient, including various diagnostic work-ups and established treatments (e.g. surgery, radiation therapy, chemotherapy, or management for various complications). When a cancer patient visits a physician, it is the physician’s responsibility to judge whether the patient’s specific complaint, and hence the medical services provided, are related to the diagnosis of his or her underlying cancer. If so, then the physician can claim for reimbursement on the category of cancer diagnosed, using the International Classification of Diseases (9th revision, clinical modification [ICD9-CM]), which is automatically registered into the database. The calculation process for the lifetime costs was as follows: The authors summed the monthly expenditures for all patients, including the cost of inpatient, outpatient and emergency care for the treatment of cervical cancer after diagnosis, and divided the aggregate by the number of these patients who were still alive during each month to estimate the monthly average costs to the NHI. Annual NHI expenditures were first adjusted to the 2010 Consumer Price Index (CPI) [21] and exchange rate (1USD = 29.322 TWDs), followed by applying a 3% annual discount rate [21,22]. The monetary value after the end of the follow-up period was assumed to be the same as the average of the last 10% of measurements through smoothing to extrapolate lifetime. The total average monthly expenditures were multiplied by the monthly survival probabilities for each stage and age group over the course of a lifetime, and all these monetary values were summed to obtain the lifetime healthcare expenditure for each group. Z-tests were also performed, with a p-value < .05 considered statistically significant.

### Uncertainties, sensitivity analysis and validation of the extrapolation method

This study used an *ex post* approach instead of the conventional *ex ante* one. Our survival data were real follow-up data for over 10 years, and the healthcare expenditures were directly retrieved from the reimbursement data files of the NHI, plus adjustment for the 2010 CPI. The authors also calculated the standard errors of the means by the bootstrap method for 100 repeated samples in these parameters, as stratified by age groups and stages, including life expectancy and EYLL, with a 95% confidence interval for lifetime healthcare expenditures.

In order to validate the extrapolation method, the authors selected sub-cohorts of patients with cervical cancer between 2002 and 2006, and then extrapolated these results to the end of 2011, and the results were compared with the Kaplan-Meier (K-M) estimates of the actual follow-ups. Assuming that the K-M estimates are the gold standard, this study calculated the relative biases for sub-cohorts with cervical cancer. The relative bias (RB) is defined as follows: RB = (estimate from extrapolation – K-M estimate)/K-M estimate.

## Results

**Figure 2:**
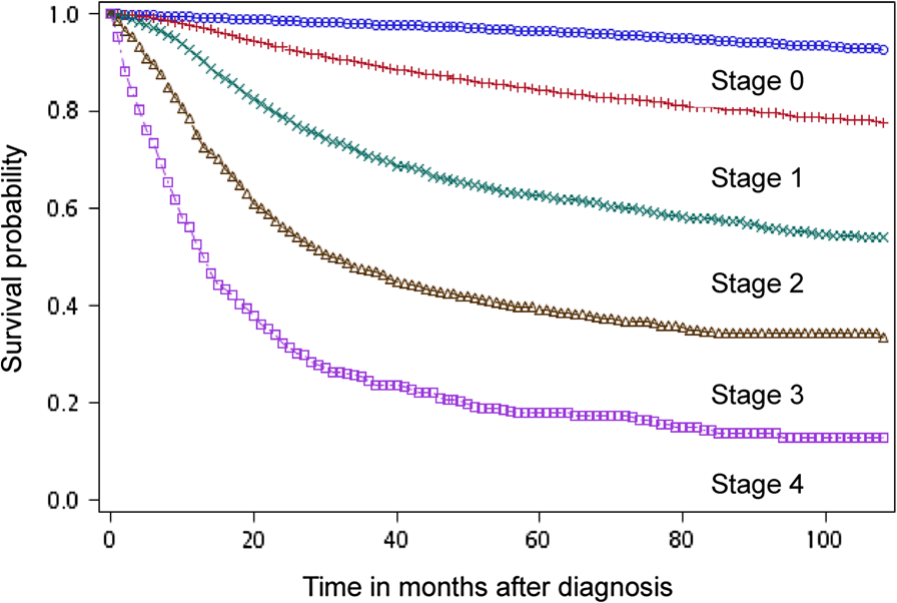
**Survival probability of cervical cancer stratified by stages.** Their five-year survival probabilities were 0.96, 0.84, 0.63, 0.39 and 0.18 in different stages from stages 0 to 4, respectively.

Cervical cancer at stages 1 to 4 had an average EYLL of 6.33 years, 11.64 years, 12.65 years, and 18.61 years, respectively; the differences among different stages were all statistically significant (z-tests, all p’s < 0.001), as shown on Table 1 and Figure 1. The mean lifetime costs of managing stage 0 (US $1,316) were found to be significantly lower than those of stages 1–4 of invasive cancer (US $7,020, $10,133, $11,120 and $10,015, respectively). The younger the age of diagnosis, the higher the EYLL (Table 1), and, in general, the earlier the stage of diagnosis, the smaller the lifetime expenditures paid by the NHI. In addition, detection of invasive cervical cancer before stage 3 compared with a more advanced stage can save life-years and costs for patients aged under 65, while those aged over 65 must be detected earlier than stage 2 to see a consistent trend with regard to these benefits (Table 1). The results obtained to validate our semi-parametric method for estimating lifetime survival show that the relative biases of extrapolation from the end of the 5th year to that of the 10th year were all below 4%, except for stage 4, due to the small sample size (Table 2). Since all values of the relative biases are negative, they indicate a trend of underestimation of lifetime survival for cervical cancer based on this method. The absolute magnitudes of such biases, however, range from 0.07 to 0.26 years, or less than 3 months. In addition, the five-year survival probabilities were 0.96, 0.84, 0.63, 0.39 and 0.18 for stages from 0 to 4, respectively (Figure 2).

**Table 1:** Life expectancy, expected years of life lost, and lifetime expenditures (USD) of cervical cancer

**Age**	**Stage**	**Case number**	Mean age at diagnosis (SD)*	**LE (SE)**^**†**^	**EYLL (SE)**^**†**^	**Lifetime healthcare expenditures (95% CI**^**‡**^**)**
All	1-4	11,096	56.46 (14.3)	19.85 (0.04)	7.78 (0.03)	8,542 (5,397-11,878)
<50 yrs,	0	10,920	38.8 (6.6)	43.47 (0.04)	-	1,087 (519–1,778)
	1	2,865	41.5 (5.6)	30.01 (0.07)	10.57 (0.06)	6,915 (4,118-9,304)
	2	737	43.3 (5.1)	19.10 (0.15)	19.86 (0.15)	11,955 (8,370-16,649)
	3	248	43.3 (5.4)	17.61 (0.23)	21.31 (0.24)	14,832 (9,755-20,478)
	4	170	42.9 (5.7)	6.55 (0.17)	32.78 (0.18)	12,069 (9,495-15,138)
50-64 yrs	0	3,923	55.9 (4.4)	27.44 (0.03)	-	1,665 (1,029-2,552)
	1	2,065	55.7 (4.3)	23.77 (0.06)	3.78 (0.05)	7,629 (4,759-11,766)
	2	1,037	56.3 (4.3)	18.51 (0.12)	8.60 (0.12)	10,599 (6,200-15,784)
	3	386	56.0 (4.3)	12.37 (0.18)	14.94 (0.18)	13,411 (9,544-18,177)
	4	248	56.3 (4.5)	5.65 (0.20)	21.43 (0.21)	12,659 (9,494-16,254)
≥65 yrs	0	2,858	72.1 (5.7)	14.28 (0.02)	-	1,725 (953–2,727)
	1	1,306	72.9 (6.5)	12.36 (0.05)	1.37 (0.05)	6,297 (3,518-9,984)
	2	1,213	74.8 (6.8)	9.02 (0.07)	3.51 (0.06)	8,511 (4,438-12,040)
	3	523	76.4 (7.4)	5.15 (0.12)	6.38 (0.12)	8,136 (4,633-10,437)
	4	298	76.5 (7.4)	2.83 (0.16)	8.61 (0.16)	6,995 (4,548-9,701)

*SD, standard deviation; ^†^SE, standard error of mean; LE, Life expectancy, in years; EYLL, expected years of life lost; ^‡^CI, confidence interval.

**Table 2:** Validation of relative bias of five-year extrapolation based on actual 10-year survival using Kaplan-Meier (K-M) estimates as the gold standard

**Stage**	**Cohort size**	Mean age at diagnosis (SD)*	**Censored rate (%)**	**10-year survival based on K-M estimate in years (SE)**^**†**^	**Extrapolation based on the first five-year follow up in years (SE)**^**†**^	**Relative bias (%)**
1	3,823	52.49 (13.20)	92.68	8.60 (0.05)	8.47 (0.02)	−1.57
2	1,791	60.39 (13.99)	80.07	6.76 (0.10)	6.69 (0.05)	−1.05
3	680	61.65 (14.91)	58.38	4.69 (0.15)	4.53 (0.08)	−3.57
4	373	60.40 (14.69)	39.68	2.71 (0.15)	2.45 (0.10)	−9.54

*SD, standard deviation; ^†^SE, standard error of mean.

## Discussion

This study is the first that simultaneously documents the improvements in life expectancy, EYLL, and savings in lifetime healthcare expenditures at different stages of cervical cancer, and the results show that in addition to stage 0, detection of cervical cancer at stages 1–3 can lead to more improvements in life expectancy and costs compared with a more advanced stage (Table 1), and the younger the age of diagnosis, the greater the benefits with regard to EYLL. However, we must carefully examine the accuracy of our estimation before making further inferences. First, since we only included patients with cervical cancer that had been verified with histopathological evidence and registered in the Taiwan Cancer Registry, the diagnoses were highly accurate. Second, because all cases of invasive cervical cancer are registered in the Catastrophic Illnesses database, the waiving of all co-payments has been under the careful monitoring and control of gyneco-oncologists, and all related reimbursements for treating cervical cancer would generally follow the established guidelines, being comprehensive and comparable for different stages. Third, all the extrapolations of survival functions are based on the validated assumption of “constant excess hazard”, which can be obtained by showing a straight line after taking the logit transform of the survival ratio between the index and age- and gender-matched referents [16,17]. As the assumption of a constant excess hazard may have a strong impact on the estimation of life expectancy for cervical cancer, we conduct a sensitivity analysis. Because the iSQoL software cannot be directly set to zero value of slope for extrapolation, we deliberately chose the second slope value that is closest to zero (either negative or positive) for extrapolation 10 years after follow-up. The results (presented in the Additional file 1) show that all the life expectancies were very close (<15% difference), indicating that our estimates are relatively accurate. Moreover, this study validated this estimation by extrapolating the survival of the first five to 10 years, and the results showed that this approach usually has less than 10% error in comparison with the actual survival based on the Kaplan-Meier method (Table 2). In one of our previous studies [7], we employed the cohort of cervical cancer patients between 1990 and 2001 in the National Cancer Registry and followed up to 2004, while the current study enrolled the cohort of 2002–2009 and followed up to 2011. Since there have been no major changes with regard to treating cervical cancer during the last two decades, it is perhaps not surprising that we found no major changes in the estimates of life expectancy between the two cohorts (19.77 years in Chu’s study versus 19.85 years in this work). However, as the life expectancy of the general female population has increased from 77.7 yrs in 1995 to 80.8 yrs in 2005, it is not unexpected that the EYLL also increased from 6.33 years to 7.78 years. Therefore, the estimation method can be seen as both consistent and accurate, and, as noted above, we tentatively conclude that detection of invasive cervical cancer before stage 3 compared with a more advanced stage can have benefits with regard to life-years and costs for patients aged below 65, while those aged over 65 must be detected earlier than stage 2 to see the same benefits. Generally speaking, the earlier the stage at diagnosis, the better the outcomes, although we might have over-estimated the effects of early detection because of potential length time bias.

Studies of cervical cancer screening tend to emphasize detection at stage 0. This study, however, provides solid evidence that detection and treatment of invasive cervical cancer at stage 1 or 2 is also very worthwhile. The calculation of EYLL in Table 1 used an age- and gender-matched general population as referents, and provides estimates for the number of life-years lost due to invasive cancer [9,12]. Because our method takes the age at diagnosis into consideration, the estimations would be less affected by lead time bias and more accurate than direct comparisons of life expectancies for cancer patients diagnosed at different stages. We recommend that the results be used to analyze the cost-effectiveness of screening programs. As Figure 2 indicates that the cervical cancer survival probabilities in Taiwan appear comparable with those reported from other countries [2-5], our findings may also be applicable to them.

### Limitations

Although this study has used the most comprehensive national data currently available in Taiwan, it has the following limitations that need to be addressed: First, the lifetime extrapolation is based on current and prior experiences, especially the national life tables; however, it is clear that such an *ex post* approach could easily underestimate the actual survival of future cancer populations, because it cannot predict the future development and adoption of newer technologies for cancer diagnosis and management. Therefore, our estimation of the lifetime survival of cancer patients may be a conservative one, while the EYLL might be overestimated. Second, because life expectancy is also a function of co-morbidity, performance states, and recurrence [23], the current estimates provide only a crude estimation of the average EYLL. Future studies with a larger cohort may stratify them into sub-cohorts based on more clinical data on co-morbidities, performance states, and recurrence, to improve the accuracy of the predictions. Third, we did not consider the growing evidence that those women who decide not to participate in screening may be inherently different from those who decide to participate, and these non-participants might have higher other-cause mortality [24]. If this phenomena occurred in Taiwan, then our EYLL would be overestimated. Fourth, this study adopted the insurer’s perspective, and only direct medical costs were estimated. Because of the lack of empirical data on the costs of out-of-pocket money or lost productivity due to cervical cancer or premature death, our results underestimate the cost of illness to the whole society. Finally, because the healthcare expenditures after the end of the follow-up period were assumed to be the same as the average of the last 10% of measurements based on kernel smoothing, this study might have overestimated the costs after the end of 10 years of follow-up. However, since almost all cases of cervical cancer would be in healthy condition 5–10 years after diagnosis, except those approaching the end of their lives, the average costs due to cervical cancer would generally become smaller given a large number of healthy survivors and higher cumulative discount rates. The potential overestimation due to this would thus be very small.

### Policy implications for community healthcare

Pap smears are not very popular among women aged 60 and older in Taiwan, which might have resulted in higher morbidity and mortality rates for cervical cancer among this group [25]. This study provides evidence that early detection of invasive cancer can saves lives and reduce costs for both young and old patients, and that the earlier detection occurs, the better (Table 1), and these facts can be used to encourage those who are otherwise afraid of undergoing cancer screening. However, further evaluations of the cost-effectiveness of this approach are needed in order to optimize the utilization of resources.

## Conclusion

Early detection of cervical cancer can save people and reduce costs for the NHI, and details of these benefits can be used to encourage regular proactive screening, especially among women older than 60, who currently are less likely to receive a pap smear in Taiwan. The prospective health benefits for patients with stages 1–3 of invasive cancer, compared to those with more advanced stages, should thus be clearly explained. The authors also recommend future studies consider evaluating the cost-effectiveness of different prevention programs, in which the likelihood of certain events (e.g., incident rates and rate ratios) can be integrated with health outcomes to improve the efficiency and fairness of the related health policies.

## Abbreviations

ICC: Invasive cervical cancer; LE: Life expectancy; EYLL: Expected years of life lost; NHI: National Health Insurance; FIGO: International Federation of Gynecology and Obstetrics; NCCN: National Comprehensive Cancer Network; CPI: Consumer Price Index; K-M: Kaplan-Meier; RB: Relative bias.

## Competing interests

The author(s) declare that they have no competing interests.

## Authors’ contributions

Conceived and designed the experiments: J-DW, Y-MC, and M-CH. Performed the data analysis: M-CH and M-TL. Wrote the paper: M-CH, J-DW and Y-MC. All authors read and approved the final manuscript.

## Authors’ information

J-DW is a Chair Professor of Department of Public Health, National Cheng Kung University College of Medicine in Taiwan. Professor Wang’s research focuses on the health services research. Y-MC is a visiting staff of Department of Obstetrics and Gynecology, National Cheng Kung University Hospital in Taiwan. Dr. Cheng’s clinical experience focuses on gynecologic oncology. M-CH is postdoctoral fellow of the Department of Public Health, National Cheng Kung University College of Medicine in Taiwan. Dr. Hung’s background includes gynecologic oncology and public health. Her research focuses on the health services research.

## Pre-publication history

The pre-publication history for this paper can be accessed here:

http://www.biomedcentral.com/1471-2407/14/505/prepub

## Supplementary Material

Additional file 1 **Sensitivity analysis of life expectancy, expected years of life lost, and lifetime expenditures (USD) of cervical cancer -Description of data: The results (presented in the Additional file**1**) show that all the life expectancies were very close (<15% difference) to our original estimates, indicating that our estimates are relatively accurate.** Click here for file
