# Supplementary material for: Estimation of savings of life-years and cost from early detection of cervical cancer: a follow-up study using nationwide databases for the period 2002–2009
Source: BMC Cancer. 2014 Jul 10;14:505. doi: 10.1186/1471-2407-14-505 (PMC4103978; doi:10.1186/1471-2407-14-505)
Supplement: Additional file 1 — Sensitivity analysis of life expectancy, expected years of life lost, and lifetime expenditures (USD) of cervical cancer -Description of data: The results (presented in the Additional file1) show that all the life expectancies were very close (<15% difference) to our original estimates, indicating that our estimates are relatively accurate. [file 1471-2407-14-505-S1.pdf]

Additional file 1. Sensitivity analysis of life expectancy, expected years of life lost, and lifetime expenditures (USD) of cervical cancer

Age	Stage	Case Number	Mean age at diagnosis (SD)*	LE (SE)†	% Differences§	EYLL (SE)†	Lifetime healthcare expenditures (95% CI‡)
All	1-4	11,096	56.46 (14.3)	19.88(0.04)	0.1	7.7(0.03)	8542 (5206-12144)
<50 yrs	0	10,920	38.8 (6.6)	43.3(0.03)	-0.4	-	1319 (766-2024)
	1	2,865	41.5 (5.6)	30.26(0.05)	0.8	10.36(0.05)	6916(4546-9752)
	2	737	43.3 (5.1)	21.96(0.13)	15	16.95(0.13)	12058 (8024-16789)
	3	248	43.3 (5.4)	17.6(0.24)	-0.1	21.31(0.25)	14831(10932-18795)
	4	170	42.9 (5.7)	7.15(0.19)	9.2	32.25(0.19)	12101(9292-15609)
50-64yrs	0	3,923	55.9 (4.4)	27.18(0.04)	0.9	-	1744 (1082-2816)
	1	2,065	55.7 (4.3)	23.78(0.07)	0.0	3.78(0.06)	7629(4427-10547)
	2	1,037	56.3 (4.3)	18.52(0.13)	0.1	8.6(0.13)	10599(6307-15173)
	3	386	56.0 (4.3)	12.41(0.17)	0.3	14.97(0.17)	13416(8858-18733)
	4	248	56.3 (4.5)	6.03(0.2)	6.7	21.08(0.2)	12661(9306-16970)
≥65 yrs	0	2,858	72.1 (5.7)	14.37(0.07)	0.6	-	1709(923-2379)
	1	1,306	72.9 (6.5)	12.61(0.08)	2.0	1.16(0.09)	6303(3286-10363)
	2	1,213	74.8 (6.8)	9.09(0.08)	0.8	3.45(0.08)	8520(4681-13314)
	3	523	76.4 (7.4)	5.18(0.12)	0.6	6.35(0.12)	8136(5448-12317)
	4	298	76.5 (7.4)	3.2(0.11)	13	8.26(0.11)	7002(4162-9246)

*SD, standard deviation; †SE, standard error of mean; LE, Life expectancy, in years; EYLL, expected years of life lost; ‡CI, confidence interval; § % difference: We re-performed extrapolation to estimate the LE with the second slope closest to zero of logit transform of survival ratio; the % difference between the two estimates was calculated using the LE of Table 1 in the original manuscript as the standard (i.e., denominator).
